# Cognitive-enhancing effects of angiotensin IV

**DOI:** 10.1186/1471-2202-9-S2-S15

**Published:** 2008-12-03

**Authors:** Paul R Gard

**Affiliations:** 1 School of Pharmacy and Biomolecular Sciences, University of Brighton, Moulsecoomb, Brighton, UK

## Abstract

Angiotensin IV is a derivative of the potent vasoconstrictor angiotensin II and it has been shown to enhance acquisition, consolidation and recall in animal models of learning and memory when administered centrally or peripherally. Whether changes in angiotensin IV activity underlie the cognitive effects of those cardiovascular drugs designed to disrupt the peripheral renin-angiotensin system in humans remains undetermined, but angiotensin IV appears to be a worthy candidate for consideration in drug development programmes. The mechanism of action of angiotensin IV is still debated, although its AT_4 _receptor has been convincingly identified as being insulin-regulated amino peptidase, which is also known as oxytocinase and placental leucine aminopeptidase. It is speculated that angiotensin IV may interact with insulin-regulated amino peptidase to enhance neuronal glucose uptake, prevent metabolism of other neuroactive peptides, induce changes in extracellular matrix molecules, or induce release of acetylcholine and/or dopamine. All of these things may be responsible for the beneficial effects on cognition, but none of them are yet proven. Importantly, strain differences in murine responses to angiotensin IV suggest that some individuals may benefit from drugs targeted to the AT_4 _receptor whilst others may be refractory. At present it thus appears that those individuals with the poorest baseline cognition may receive greatest benefit, but possible genetic differences in responses to angiotensin IV cannot be ruled-out.

## Background

Using object recognition as a model of memory and learning, we have shown that angiotensin IV causes significant enhancement in mice. The object recognition test involves exposing the animals to an open field (60 × 40 cm) in which are placed two identical ethanol-cleaned porcelain objects that are novel to the mice, and of sufficient weight that they cannot be moved or displaced by the subjects. The mice are allowed 3 minutes to explore the field and the objects, before being returned to their home cage. One hour later they are again placed into the same field, with the same objects, all ethanol-cleaned, and again allowed 3 minutes to explore. At the conclusion of the second training period the mice are injected subcutaneously with angiotensin IV and returned to their home cage.

Twenty-four hours later, the animals are returned to the same field, but now one of the porcelain objects has been replaced by one of a different shape and colour, but again ethanol-cleaned. The time spent by the mouse in exploring the novel object, in comparison to the familiar object, and taking into account general exploratory/locomotor activity, is taken as a measure of learning and memory ('cognition'). In DBA2 strain mice, saline-treated animals spent approximately 10% more time exploring the novel object in comparison to the familiar object. Mice treated with angiotensin IV, at a dose of 0.47 mg.kg^-1 ^spent approximately 40% more time exploring the novel object (*p *< 0.05) [1].

This was not the first demonstration of an effect of angiotensin IV on learning and memory. Braszko *et al. *[2] had reported that intracerebroventicular (icv) administration of 1 nmol of angiotensin IV (0.78 μg) to rats significantly enhanced recall of a passive avoidance task, and significantly enhanced acquisition of an active avoidance task. A similar study was reported in 2006 by the same group [3] in which angiotensin IV was given at the same dose and by the same route to rats 5–15 minutes before recall of a passive avoidance task and recall of an object recognition task. The results indicated that angiotensin IV significantly enhanced recall. Another group of workers (Wright, Harding and colleagues), reported independently that norleucine-angiotensin IV, a metabolically stable analogue of angiotensin IV, at 1 nmol icv, enhanced recall of a passive avoidance task [4] and reversed the amnesic effect of scopolamine by enhancing acquisition in a spatial memory task (Morris water maze) [5].

The work of the groups of Braszko, Harding, and Wright indicate that angiotensin IV, or its analogue, delivered directly to the brain, can enhance recall and acquisition in rats. Our work demonstrates that peripherally administered angiotensin IV can enhance memory consolidation in mice. These disparate findings illustrate the wide-reaching effects of angiotensin IV on learning and memory, and the possibility of peripheral administration increases its therapeutic potential.

**Figure 1:**
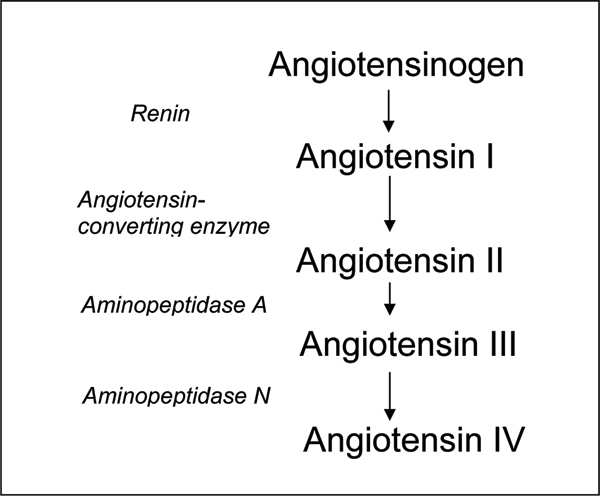
Synthetic pathway of the angiotensins.

**Figure 2:**
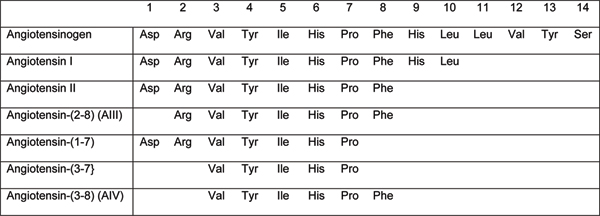
Primary structures of members of the angiotensin family of peptides.

Angiotensin IV is a component of the renin-angiotensin system, a metabolic product of the potent vasoconstrictor angiotensin II (Figures 1 and 2). The significance of this is that any therapeutic regime aimed at reducing the effects of angiotensin II on the cardiovascular system may also alter synthesis of angiotensin IV, with potential knock-on consequences for cognition. Currently, several therapies for hypertension and heart failure act via the renin-angiotensin system to prevent the vasoconstrictor actions of angiotensin II. Angiotensin-converting enzyme (ACE) inhibitors, prevent the conversion of angiotensin I to angiotensin II by ACE. Antagonists of the angiotensin type 1 (AT_1_) receptor (angiotensin II antagonists (AIIAs); angiotensin receptor blockers) selectively prevent the vasoconstrictor actions of angiotensin II. More recently, renin inhibitors, for example, aliskiren, which prevent the synthesis of all of the angiotensins, have been launched. Intuitively, with the possible exception of the AT_1 _receptor antagonists, all of these therapies might be predicted to decrease synthesis of angiotensin IV, and thus have detrimental effects on learning and memory. That, however, disregards the fact that alternative enzymatic pathways exist that may allow the synthesis of the smaller, later angiotensin peptides despite blockade of enzymes like ACE [6], and that the brain possesses a complete renin-angiotensin system that may be affected differently by therapeutic agents with limited ability to cross the blood brain barrier than the peripheral renin-angiotensin system [7]. The evidence in fact indicates that ACE inhibitors, and AIIAs, when given acutely to cognitively healthy volunteers, are able to improve performance on a battery of psychological tests [8,9]. Furthermore, chronic treatment with these agents has been suggested to have beneficial effects on cognition in the elderly. A review by Amenta and colleagues [10] suggests that the treatment of essential hypertension with ACE inhibitors produces positive cognitive outcomes that are significantly better than those induced by either diuretics or beta-blockers, although all are effective in regulating hypertension. The Study on Cognition and Prognosis in the Elderly (SCOPE) reported that treatment of hypertension with the AIIA candesartan resulted in less cognitive decline than that observed in the untreated control group [11]. Also looking at another AIIA, Tedesco and colleagues [12] undertook a 26-month trial comparing losartan with a diuretic in matched samples of older (60–73 years) and younger (30–59 years) mild-to-moderate hypertensives. They reported an absolute improvement in cognition in both groups receiving losartan, paralleled with reduced systolic and diastolic blood pressure, and significant improvements in quality of life scores. Blood pressure and quality of life scores also improved significantly for the diuretic groups, but there were no parallel improvements of the cognitive measures. More recently, Fogari and colleagues reported in three separate studies that the AIIAs losartan, valsartan, and temisartan produced positive effects on cognition in very elderly (aged 75–89 years) mild-to-moderate hypertensives, with significant advantages on episodic memory after 12–24 week treatment programs [13-15]. The mechanism underlying the cognitive-enhancing effects of the ACE inhibitors and the AIIAs is unknown, but there is evidence that manipulation of the renin-angiotensin system may improve cognitive performance independent of changes in haemodynamics and protection against vascular damage. Whether any beneficial effects of ACE inhibitors and AIIAs involve perturbation of angiotensin IV activity, and whether drugs targeted at angiotensin IV might have greater therapeutic potential, remains speculation.

The actions of angiotensin IV and its physiological significance is still an enigma. It is now clear that the brain possesses a complete renin-angiotensin system and that it is involved in a range of physiological activities, including control of fluid balance and blood pressure [16]. More importantly, the locations of the receptors for the angiotensins have now been mapped within the brain. Early studies concentrated on the areas poorly protected by the blood brain barrier because of the relative inability of peripheral angiotensin to cross. Accordingly, AT_1 _receptors were identified in the circumventricular organs (subforniculate organ, organum vasculosum of the lamina terminalis, area postrema, median eminence, and anterior pituitary gland). Since then, more receptors have been located in areas within the blood brain barrier, including supraoptic, preoptic, and ventral medial nuclei [6]. Angiotensin type 2 (AT_2_) receptors are found in areas distinct from AT_1 _receptors, such as the globus pallidus, inferior colliculus, locus coeruleus and thalamus [6]. AT_4 _receptors, which are specific for angiotensin IV, are similarly widely distributed within the brain and are sometimes in areas also possessing AT_1 _or AT_2 _receptors, for example, the anterior pituitary, caudate puatamen, globus pallidus, and thalamus, but also in the hippocampus, which is devoid of the other angiotensin receptors [6]. It is this localisation of AT_4 _receptors to the hippocampus that is most interesting in terms of angiotensin IV effects on learning and memory.

The location of the AT_4 _receptors is well understood, but the nature of the receptor remains a matter of controversy. The AT_4 _receptor was first identified in bovine adrenal membranes by Harding, Wright, and co-workers [17], and subsequently in other mammalian tissues, for example, the brain, kidney, bladder, colon and uterus. It was later determined to be trimeric and receptors from different anatomical locations were found to be of equivalent molecular weights [18]. This suggested that there are no markedly different subtypes, or that any subtypes were of a very similar structure. Importantly, it was apparent that the receptor was not a typical 7-transmembrane domain G-protein coupled receptor [19]. The landmark finding came in 2001 when it was discovered that the AT_4 _receptor was an insulin-regulated aminopeptidase (IRAP) [20]. The finding was based on sequence homologies of fragments of the human brain AT_4 _receptor and IRAP, and similarities of the molecular weights of the two binding sites. Expression of cloned IRAP in cultured cells yielded a receptor with binding characteristics almost identical to those of the native AT_4 _receptor. Albiston has since argued persuasively that IRAP is the physiological receptor for angiotensin IV, and that there are several possible mechanisms by which angiotensin IV could be acting to enhance memory [21]. To summarise the argument: the AT_4 _receptor and IRAP have identical binding properties for angiotensin IV, and expression of the cDNA for IRAP gives rise to a protein with the binding characteristics of the AT_4 _receptor. IRAP and its mRNA are co-localised in the brain with AT_4 _receptors as determined by radioligand binding. The endogenous peptide LVV-Hemorphin-7, which is structurally unrelated to angiotensin IV but known to bind to AT_4 _receptors, inhibits the enzymatic activity of IRAP *in vitro*, similar to angiotensin IV.

IRAP is a member of a group of enzymes that includes aminopeptidases A and N, both of which are involved in the metabolic cascade of the renin-angiotensin system. Originally identified in adipocytes and skeletal muscle cells co-localised with the insulin-regulated glucose transporter GLUT4, it is also referred to as oxytocinase or placental leucine aminopeptidase. In the presence of insulin, IRAP and GLUT4 translocate to the cell membrane where GLUT4 mediates glucose uptake. GLUT4 has been identified in the brain in regions associated with AT_4 _receptors and it has been speculated by Albiston that inhibition of IRAP by angiotensin IV might increase glucose uptake into brain cells resulting in facilitation of learning and memory. There is independent evidence that glucose enhances processes associated with learning and memory [22].

IRAP is also known to hydrolyse angiotensin III, oxytocin, vasopressin, met-enkephalin, and other endogenous peptides. Some of these peptides, for example, oxytocin and vasopressin, have been implicated in learning and memory, and it also has been suggested by Albiston *et al*. [21], that inhibition of IRAP by angiotensin IV results in the accumulation of these peptides that enhance memory.

Wright and Harding [6], however, argue with equalled cogency that inhibition of IRAP cannot explain the cognitive effects of angiotensin IV. The salient points of their argument are: the effects of enzyme inhibition and accumulation of endogenous peptides are slow, in the order of hours or days, whilst the onset of action of angiotensin IV in some tissues is within seconds; the concentrations of angiotensin IV required to produce biological, that is, cognitive effects, are well below the concentrations required to inhibit IRAP; and there is dispute as to whether angiotensin IV is a competitive substrate of IRAP or whether it binds allosterically. If it is a competitive substrate, it would be difficult to explain the observed effects of the AT_4 _receptor antagonist divalinal, which is known to not only block the effects of angiotensin IV, but also have detrimental effects on learning and memory: does divalinal enhance IRAP activity?

The compromise suggested by Wright and Harding [6] is that angiotensin IV binds to IRAP, its cognate receptor, but that there is some other transduction mechanism not reliant on inhibition of the enzyme. They attempt to integrate the knowledge of the actions of angiotensin IV and the changes in extracellular matrix molecules associated with learning and memory, but as yet an unequivocal mechanism has not been elucidated.

A further suggestion concerning the mechanism of action of angiotensin IV involves enhancement of long-term potentiation and acetyl choline release in the hippocampus (see [23] for a review). Braszko [24,25] has produced evidence that dopamine D1 and D2 receptors are involved in the cognition-enhancing effects of angiotensin IV. Whatever the mechanism, it is clear that angiotensin IV enhances several components of learning and memory, although this effect does not appear to be consistent for all individuals.

**Figure 3:**
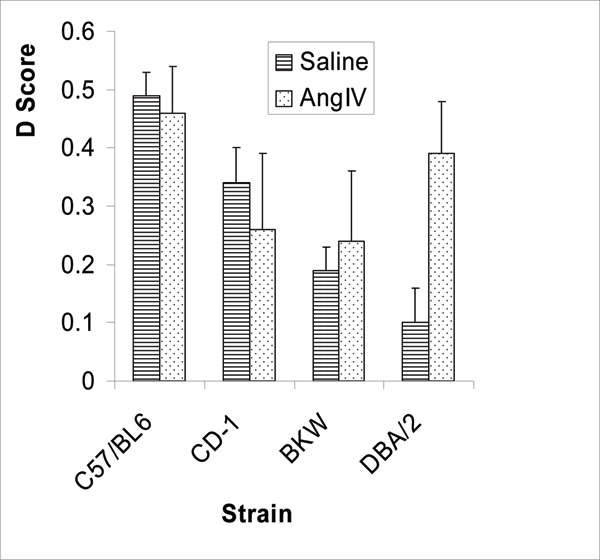
Strain differences in the effects of angiotensin IV (AngIV; 0.47 mg.kg^-1^, subcutaneous) on object recognition in the mouse. The D score represents differential exploration of a novel object in comparison to a familiar object. Significant difference between vehicle control and angiotensin IV for DBA2 mice only, *p *< 0.05. (Adapted from [1].)

Our laboratory has previously studied the effects of angiotensin II on behaviour, and specifically, the behavioural effects of the AIIA losartan. In mice, losartan produces anxiolytic-like and antidepressant-like effects, and, in some cases, improved learning and memory (see [7] for a review). Importantly, not all strains of laboratory mouse exhibit the same behavioural responses to losartan, with BKW strain mice being particularly sensitive to its anxiolytic effects [26]. Furthermore, there is evidence of an under-active renin-angiotensin system in these mice [26]. Strain differences in the behavioural effects of peripherally administered angiotensin IV were therefore investigated using the object recognition test described above in an attempt to identify correlations between the behavioural effects of an AIIA and those of angiotensin IV. Figure 3 illustrates the strain differences in the behavioural effects of angiotensin IV and shows that DBA2 mice are more responsive to the effects of angiotensin IV than the BKW mice identified in the losartan studies [1]. The interesting feature of these results is that the mice with best inherent cognition benefit least from angiotensin IV. This either indicates a 'ceiling effect' of the behavioural method, that is, the inability to measure greater cognition, or the possibility that angiotensin IV replaces some deficit responsible for the inherent poor performance.

The remainder of this review will concentrate on the possible interaction of angiotensin IV, IRAP, also known as oxytocinase and oxytocin. In rats and mice, low doses of oxytocin improves social memory, that is, recognition of conspecific animals [27,28]. That oxytocin is inherently involved in this form of memory is demonstrated by the fact that in oxytocin-knockout mice there is a deficit in social memory that can be restored by icv oxytocin [29]. In normal animals, however, higher doses of oxytocin disrupts social memory [30]. Similarly, high levels in mice reduce conditioned avoidance [31] and learned helplessness [32]. If angiotensin IV produces its effects via interaction with IRAP and oxytocin, a similar pattern of effects might therefore be seen. Our experiments studied the interaction of angiotensin IV both *in vitro *and on the behaviour of BKW mice. The results showed that in the isolated mouse uterus, angiotensin IV induces contractions, which can only partially be blocked by the AT_1 _receptor antagonist losartan, indicating some non-AT_1 _receptor-mediated effect. Furthermore, it potentiated the contractile effects of oxytocin, suggesting inhibition of IRAP (oxytocinase) [33]. In the forced swim test, however, the amnesic effect of oxytocin on learned helplessness was reduced by angiotensin IV. These results may be interpreted as an indication that by inhibiting IRAP (oxytocinase), angiotensin IV increases endogenous oxytocin to concentrations in the amnesic range, or that angiotensin IV blocks the behavioural effects of oxytocin by some other mechanism. Our work, in combination with that of others, suggests that it is the peptide metabolites of oxytocin that are responsible for the enhanced cognition, and that angiotensin IV prevents its effects by inhibiting IRAP (oxytocinase) and the metabolism of oxytocin. The conclusion is that oxytocin is unlikely to be involved with the cognitive actions of angiotensin IV, and that the resistance of BKW strain mice to the cognitive effects of angiotensin IV is unlikely to be due to an inherent disorder of oxytocin.

## Conclusion

This area of research is still in its infancy. It is known that angiotensin IV can improve cognition through effects on acquisition, consolidation, and recall, and that it is possible to elicit these effects following peripheral administration of the peptide. Drugs known to interfere with the renin-angiotensin system have been shown to have beneficial cognitive effects in humans, but whether these effects involve angiotensin IV is unclear. It is possible, however, that not all individuals will show similar cognitive responses, as strain differences in mice have been identified, suggesting either genetic differences in the effects, replacement of some deficit, or some other ceiling effect. This may mean that, should an angiotensin IV analogue be developed for the treatment of cognitive deficits, not all patients would benefit from their use. Importantly, the mechanism of action of angiotensin IV remains unresolved, although inhibition of IRAP, and consequent increases in endogenous peptides such as oxytocin, currently seems unlikely.

## List of abbreviations used

ACE: angiotensin-converting enzyme; AIIA: angiotensin II antagonist; AT_1_: angiotensin type 1 receptor; AT_2_: angiotensin type 2 receptor; AT_4_: angiotensin IV receptor; icv: intracerebroventicular; IRAP: insulin regulated amino peptidase.

## Competing interests

The author is currently in receipt of research funding from the Institute for the Study of Aging (ISOA) for the preliminary investigation of novel drugs targeted at the angiotensin IV receptor.
